# The impact of early weight-bearing on results following anterior cruciate ligament reconstruction

**DOI:** 10.1186/s12891-024-07525-8

**Published:** 2024-05-21

**Authors:** Sehmuz Kaya, Yunus Can Unal, Necip Guven, Can Ozcan, Abdulrahim Dundar, Tulin Turkozu, Sezai Ozkan, Cihan Adanas, Mehmet Ata Gokalp

**Affiliations:** 1 Department of Orthopaedics and Traumatology, Yuzuncu Yil University, Van, Turkey; 2 Department of Orthopaedics and Traumatology, Orhaneli State Hospital, Bursa, Turkey; 3 Department of Orthopaedics and Traumatology, Hitit University, Corum, Turkey

**Keywords:** Anterior cruciate ligament, Arthroscopic reconstruction, Rehabilitation, Early weight bearing, Body mass index

## Abstract

**Introduction:**

Anterior cruciate ligament (ACL) ruptures are common injuries that typically affect young, physically active individuals and may require surgical reconstruction. Studies have shown that the long time success of ACL reconstruction depends on the surgical technique and the postoperative rehabilitation strategy. However, there is still no consensus on the content of rehabilitation programs. Hence, additional research is required to elucidate the significance of early weight-bearing in the rehabilitation process following ACL reconstruction. The aim of this article is to examine the impact of weight-bearing on the clinical results of ACL reconstruction.

**Materials and methods:**

We retrospectively reviewed patient records who had undergone arthroscopic reconstruction using a semitendinosus-gracilis tendon graft for anterior cruciate ligament rupture between January 2018 and December 2020. The study included the data of 110 patients. The patients were split into two groups: Group 1 underwent early weight-bearing, while Group 2 followed a non-weight-bearing regimen for three weeks. We assessed the patients using the anterior drawer test, Lachman test, range of motion, Lysholm knee scale, Cincinnati scale, Tegner scale, International Knee Documentation Committee (IKDC) form and clinical records. Analytical tests were conducted to compare the results.

**Results:**

The complication rates did not show a significant difference between the groups. Group 1 had higher frequencies of positive anterior drawer and Lachman tests. The Lysholm and Cincinnati knee scores of patients in Group 1 were notably lower than those of patients in Group 2. Additionally, the Tegner activity scores and IKDC scores of patients in Group 1 were also meaningfully lower than those of patients in Group 2. In Group 1 patients, there was no notable relationship observed between body mass index (BMI) and the results of the anterior drawer test (ADT) or Lachman test. However, patients with a BMI of 25 or higher in Group 1 showed a decrease in postoperative IKDC scores. In Group 2 patients, no significant relationship was identified between BMI and either the ADT or the Lachman test outcome.

**Conclusion:**

Based on current literature and current rehabilitation guidelines following ACL reconstruction, the decision to initiate early weight-bearing is based on a limited number of studies with low levels of evidence. In our study, we found that patients who followed a non-weight-bearing regimen for 3 weeks after surgery had better mid-term results than those who were allowed to bear weight early. It appears that further prospective studies on this topic are needed to update rehabilitation guidelines in the next.

## Introduction

An anterior cruciate ligament (ACL) tear is a widespread injury that typically affects young, physically active individuals and requires reconstruction [1]. In the United States alone, it is estimated that more than 200,000 new ACL injuries occur every year [2]. The long-term effectiveness of anterior cruciate ligament reconstruction depends on the chosen method of reconstruction and the postoperative rehabilitation process. Several factors can influence the success of the surgery and rehabilitation, including the patient’s age, physical activity level, body mass index, extent of cartilage damage, range of motion limitations in the knee joint, muscle atrophy, presence of effusion, pain, time elapsed between injury and surgery, smoking habits, and whether the patient underwent preoperative rehabilitation. These factors can contribute to a more successful postoperative rehabilitation process for patients [3].

A technically flawless reconstruction may have negative outcomes due to inadequate and inappropriate rehabilitation [4]. Considering that % 35 of athletes do not return to their preinjury sports level within two years after anterior cruciate ligament reconstruction and that %35 of patients develop symptomatic tibiofemoral osteoarthritis in the 10 years after surgery, effective anterior cruciate ligament reconstruction and postoperative rehabilitation are of the utmost importance [3, 5]. However, there is no agreement on the best strategy or assessment method for rehabilitation programs [6]. This can be confusing for both patients and physiotherapists [7]. A study reviewing six high-quality publications outlining rehabilitation guidelines after anterior cruciate ligament reconstruction found different results regarding the impact of early full weight-bearing on clinical outcomes. This highlighted the need for clearer definitions of accelerated rehabilitation and early load bearing [8]. Unfortunately, the rate of failed ACL reconstruction requiring reoperation has been reported to be between % 10 and %15 [9]. Aggressive rehabilitation protocols performed before biological integration and complete ligamentization can cause bone tunnel widening due to exposure of the graft-bone interface to early stresses [10]. Based on our clinical observations and literature review, we found that there is no consensus on weight bearing after anterior cruciate ligament reconstruction. Therefore, the aim of our study was to address this confusion by investigating the medium-term effects of early weight-bearing compared to non-weight-bearing on the clinical outcomes of rehabilitation following anterior cruciate ligament reconstruction surgery.

## Materials and methods

We analysed the records of 353 patients who underwent reconstruction with semitendinosus-gracilis tendon grafts for ACL rupture between January 2018 and December 2020. Patients who used braces in the postoperative period, patients with concomitant injuries and patients who did not attend the follow-up appointment were excluded from the study. Patients with isolated anterior cruciate ligament injury were included in the study. The number of patients included in the study was 110 and all operations were performed by 3 surgeons.

**Fig. 1:**
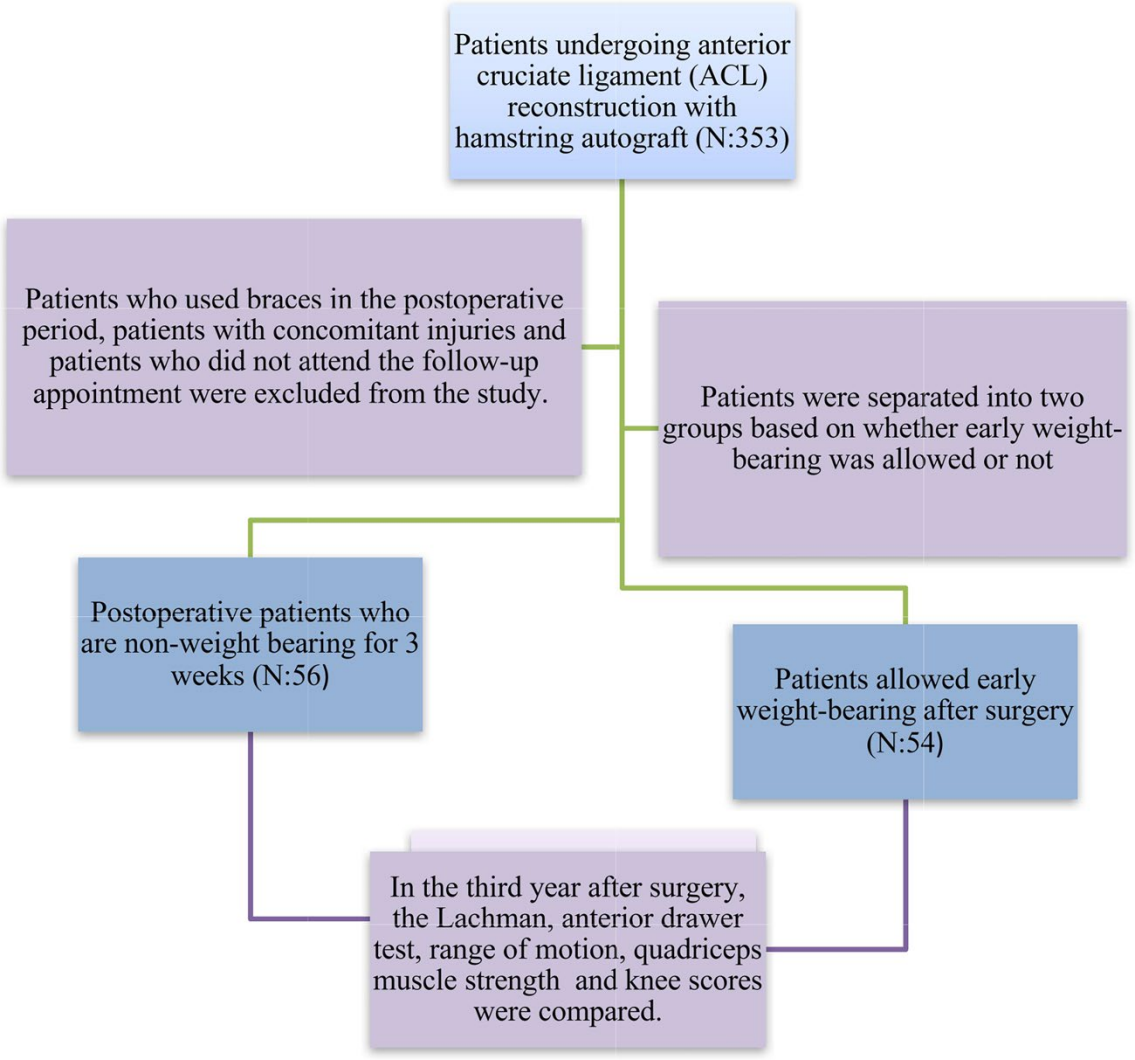
Participant flow chart

The patients were categorized into two groups: Group 1 consisted of 54 patients who were allowed early weight-bearing on the 1st day of the postoperative follow-up period, while Group 2 consisted of 56 patients who were not allowed to non-weight-bearing for 3 weeks. Neither group received preoperative rehabilitation. The results and scores of the anterior drawer test, Lachman test, range of motion, Lysholm knee scale, Cincinnati scale, Tegner scale and International Knee Documentation Committee (IKDC) form were obtained from the patients’ clinical records and control examinations performed by 2 experienced surgeons (Fig. 1). The quadriceps muscle strength of the patients was measured with a hand dynamometer by the same surgeons. In addition, perioperative tibial slope was measured.

### Surgical technique, medication, and rehabilitation

All patients underwent arthroscopic ACL reconstruction using a tourniquet. Preoperative antibiotic prophylaxis and postoperative low molecular weight heparin prophylaxis were administered. The knee joint was evaluated by opening standard arthroscopy portals. Gracilis and semitendinosus tendons were removed using a tendon stripper. The femoral tunnel was opened in the appropriate position according to the anatomical side and in the appropriate thickness for the graft. The tibial tunnel was opened at a 55-degree angle to the tibial plateau.

The prepared grafts were fixed using a single bundle fixed-loop device in the femur and absorbable screws and staples in the tibia. Drains were used in all patients and removed on postoperative day 1.

Patients in group 1 were given as much weight as they could tolerate the pain with crutches on postoperative day 1. In the following days, the amount of weight bearing was increased and full weight bearing without support was achieved on postoperative day 5. Patients in group 2 were mobilised with crutches on postoperative day 1 and non weight bearing was performed until postoperative day 20. On the 21st postoperative day, the amount of weight bearing was increased and full weight bearing was achieved on the 25th postoperative day. The same rehabilitation programs were applied for both groups except for the weight-bearing protocol. Swelling management was done with Ice, compression and elevation. In both groups, we started open chain exercises from postoperative day 1, full extension and flexion up to 90 degrees were allowed for the first 3 weeks and full flexion was allowed after the 3rd week. After the 3rd postoperative day, they were allowed to lift 1 kg weight and increase weight by 1 kg every 3 days and we also applied a home exercise program for rehabilitation.

### Statistical analysis

All data were analysed using SPSS version 26 after coding. The Kolmogorov‒Smirnov test and histogram graphs were utilized to assess whether normal distribution conditions were met. For variables that exhibited normal distribution, the mean and standard deviation values were taken into consideration. For nonnormally distributed parameters, the median and minimum-maximum values were provided. Percentage and ratio values were calculated for nominal and categorical variables. The relationship between the variable of early load or not and the analysis of complications was performed using the chi-square test. Similarly, the Mann‒WhitneyU test was employed to compare the scores with the variable of giving or not giving early load. Two way ANOVA was used in the comparisons of “group 1-group 2” and “BMI groups”. Following this analysis, “Bonferroni post-hoc test” was used to determine the groups making the difference for each score. A statistical significance level of %5was accepted.

### Findings

The study included 110 adult patients who underwent anterior cruciate ligament reconstruction. The number of patients in group 1 was 54 and 56 in group 2. Of these patients, 109 (% 99.1) were male. The median age of the patients in Group 1 was 31.5 years, while in Group 2, it was 28.8 years. Due to some technical problems, the operation time was prolonged in a few cases, the mean operation time was 75 min (range: 65–110 min). Table 1 presents the demographic data.

**Table 1:** Demographic data table of the patient

	Group 1	Group 2
Number of Patients	54	56
Age	31.5	28
Gender (Male/Female)	53/1	56/0
Side (Right/Left)	26/28	32/24
BMI	25.8	26.7

Tibial slopes of patients in group 1 7.60 ± 0.80, tibial slopes of patients in group 2 7.80 ± 0.79 and there was no statistically significant difference (*p* = 0.782).

There was no notable difference in complication rates between Group 1 and Group 2 (*p* = 0.345). These complications included haemarthrosis, rerupture, infection and deep vein thrombosis. In group 1, 4 patients had rerupture, 2 patients had infection and 1 patient had dvt, whereas in group 2, 3 patients had haemarthrosis, 3 patients had rerupture and 1 patient had infection. Comparisons were made between the postoperative results of the anterior drawer tests (ADT) and Lachman tests of patients in Group 1 and Group 2. It was observed that Group 1 patients had positive anterior drawer tests more frequently than Group 2 patients, which was statistically significant (*p* = 0.024). Similarly, Group 1 patients had positive Lachman tests more frequently than Group 2 patients, which was also statistically significant (*p* = 0.013). When analysing the Lysholm knee scores of patients in both groups, there was no significant difference in the preoperative Lysholm score (*p* = 0.811). However, the postoperative Lysholm knee scores of Group 1 patients were significantly lower than those of Group 2 patients. The score was 46.67 in Group 1 and 64.02 in Group 2 (*p* = 0.004). The Cincinnati knee scores of patients in both groups were not significantly different preoperatively, but postoperatively, Group 1 patients had significantly lower scores (*p* = 0.003). By comparing the postoperative Tegner activity score between the two groups, it was found that Group 1 patients had a significantly lower Tegner score than Group 2 patients (*p* < 0.001). Similarly, when comparing the postoperative IKDC score, Group 1 patients had significantly lower scores than Group 2 patients (*p* = 0.005). There was no significant difference in range of motion between the two groups. When we looked at the quadriceps muscle strength of the patients, it was 127,78 *N* ± 11,962 in group 1 and 127,14 *N* ± 13,172, in group 2 and it was not statistically significant (*p* = 0.792).

In Group 1, the ADT and Lachman test results were analysed using chi-square analysis based on BMI categories: <25 and ≥ 25. There was no significant correlation observed between the ADT and BMI (*p* = 0.285), as well as between the Lachman test and BMI (*p* = 0.458). Similar findings were observed in Group 2, where no difference was found between the BMI and ADT (*p* = 1) or between the BMI and Lachman test (*p* = 0.573).

When the results of the two-factor comparison were analysed, the interaction effect of early weight bearing and BMI on the Lysholm score was not significant (*p* = 0.134). While the interaction was not significant in Cincinati and Tegner scores (*p* = 0.093/*p* = 272), the interaction was significant only in IKDC score (*p* = 0.048).

## Discussion

Anterior cruciate ligament (ACL) reconstruction is a frequently performed procedure to help patients return to their active lifestyle. Proper rehabilitation of the reconstructed knee is crucial for patients to return totheir previous level of physical activity. Many randomized studies have been conducted to assess the effectiveness of early weight-bearing versus non-weight-bearing after ACL reconstruction [11]. Early studies on early weight-bearing after ACL reconstruction reported that it reduced anterior knee pain and improved Lysholm scores. However, it is important to note that these studies involved patients who were evaluated at an average of 7 months after undergoing reconstruction with a patellar tendon graft, which may explain the difference in our study results [12]. A systematic review on rehabilitation after ACL reconstruction in 2016 concluded that early weight-bearing reduced anterior knee pain and did not increase the risk of tunnel widening [3]. According to the rehabilitation guidelines of the Multicentre Orthopaedic Outcomes Network (MOON) group, postoperative early weight-bearing is recommended [4]. However, it is worth noting that this recommendation is based on a single study conducted in 1998. In our study, we found that early weight-bearing increased knee laxity and decreased knee scores. Based on these findings, it appears that further studies are needed to inform updates to rehabilitation guidelines.

Even studies conducted on living subjects to develop rehabilitation programs that do not strain or restrict the healing of the ACL graft have not yielded conclusive results regarding the impact of loading on ACL healing [13]. Publications that highlight the positive outcomes of early motion and weight-bearing also acknowledge the uncertainty surrounding the safe threshold of loading. The debate about which rehabilitation approaches are too aggressive and what level of loading is sufficient persists [14]. This uncertainty may expose patients to anunnecessary risk of reinjury. Our study’s findings, which show superior scores among patients who did not undergo non-weight-bearing, can contribute to resolving this debate. Clinicians can achieve satisfactory clinical outcomes by refraining from applying weight-bearing to patients for 3 weeks and closely monitoring them during the postoperative rehabilitation process, without concern about the patient experiencing a recurrence of ACL insufficiency. In a publication comparing the clinical and radiological outcomes of patients following a non-weight-bearing regimen for 1 week compared with 2 weeks during the postoperative rehabilitation period, no significant differences were found in Tegner activity level, Lysholm score, anterior laxity, or muscle strength values. However, the same study reported that early weight bearing could potentially increase the risk of femoral tunnel enlargement [15]. In light of these factors, it would be prudent to avoid implementing aggressive rehabilitation protocols that carry the risk of potential reinjury until the literature provides clearer guidance on this matter. Another study demonstrated that aggressive rehabilitation led to increased femoral tunnel enlargement and decreased IKDC scores, emphasizing the importance of a balanced approach to rehabilitation [16]. This study emphasised the importance of early mobilisation and rehabilitation to restore normal range of motion after surgery and also observed the positive effects of non-weight bearing on outcomes. In the literature, there are inconsistent and conflicting findings about whether the patient’s BMI is a risk factor for the development of anterior cruciate ligament insufficiency after reconstruction [17]. In our study, we found that one of the scores decreased but the other scores did not change.

## Conclusion

In the current literature, it has been observed that according to rehabilitation guidelines after ACL reconstruction, the decision to begin early weight-bearing is based on low level evidence in a limited number of studies. Our study shows that patients who were not allowed to bear weight for 3 weeks after ACL reconstruction had better results in the mid-term compared to those who started early weight-bearing. Additionally, we found that early weight-bearing in overweight patients led to a greater decrease in knee scores. Therefore, it is important to exercise caution when considering early weight-bearing in the overweight patient group. Future updates to rehabilitation guidelines should include evidence from prospective and more comprehensive studies on early weight bearing.

### Restrictions

The retrospective nature of our study is our first limitation. Another limitation of our study may be the lack of preoperative whole knee scores, quadriceps strength data and knee alignment for all patient groups. Another factor is that radiographic imaging methods were not used in our study. More powerful studies can be done by investigating the correlation between clinical scores and radiographic findings. A larger number of patients in the groups would have increased the power of the study. Another limitation is that not all operations were performed by a single surgeon. This may create the possibility that surgeons may affect the results by using a different technique.

## Data Availability

The datasets used and/or analyzed during the current study are available from the corresponding author on reasonable request.
